# Pure Phosphorus in Diseases of the Nervous System

**Published:** 1891-08

**Authors:** Bat Smith

**Affiliations:** Columbus, Texas


For Daniel's Texas Medical Journal.

### PURE PHOSPHORUS IN THE DISEASES OF THE NERVOUS SYSTEM.

BY BAT SMITH, M. D., COLUMBUS, TEXAS.

A DISTINGUISHED author has truly said "The tendency of the times is to precision and accuracy;" and, indeed, is he a bold man, who, in this day, would advance an hypothesis, without being able to demonstrate his theory, well supported by facts. Recognizing the fallacies which might lead to erroneous deductions from theories too dogmatic in character, it is not the intention of the writer to enter upon a discussion relative to the general properties of phosphorus, either as a chemical or therapeutic agent. I only ask a careful consideration of the few facts as they appear below.

The following cases came under my observation some years ago; and, while my experience with phosphorus is yet too limited to determine with accuracy its value, as compared to other therapeutic agents, yet the results obtained in these instances may furnish some data which may prove of interest.

CASE I. A lady, aged about 35 years. The family history gave no evidence of tuberculosis or scrofula. For thirteen years she had suffered from locomotor ataxia. She complained of no pain whatever, there being simply a gradual wasting of the gluteal muscles, and especially of the flexor muscles of the legs, in particular the biceps, semi-membranosus and semi-tendinosus. She had a gliding motion, but was unable to lift her foot from the floor. Upon careful examination I found no sugar in the urine, of albumen scarcely a trace; the phosphates, however, were in larger quantities, and the phosphoric acid passed in twenty-four hours in an enormous amount, considering the weight of the patient (92 pounds). The phosphoric acid passed in the twenty-four hours varied from 48 to 56 grains. Her appetite was fair, and digestion seemed to be good. She assured me she had been under treatment for some years without benefit, and that her powers of locomotion were becoming worse from day to day.

She was put upon a treatment of pure phosphorus, one-twentieth of a grain dissolved in oil of sweet almonds, 3 times daily; also 1-10 of a grain of the solid extract of belladonna was given morning and night. At the end of two weeks the belladonna was suspended and the phosphorus continued. About four weeks from the beginning of the treatmant the muscles seemed gradualy to enlarge, and the motion of the legs became much freer. The improvement continued, and at the end of six weeks the dose of phosphorus was diminished from 1-20 to 1-30 of a grain. Shortly after this the drug was administered only twice daily, morning and night. At the end of six months the patient had so far recovered that she could get up and down stairs without difficulty. Ten months from the commencement of the treatment she had recovered the entire use of her legs, being able to walk as well as ever. Her weight had increased from 92 to 126 pounds. Two years afterwards she wrote to me, stating that she feared a return of her former affliction, and asking that I should send her some more of the medicine. She was placed under the same treatment, and the results were entirely satisfactory.

CASE NO. II. A Mr. S., 25 years, applied to me for some bromide of potassium, stating that he was subject to epilepsy, and that this drug alone gave him relief. Upon inquiring into the cause of these epileptic attacks I learned from him that he had had a sun stroke when he was ten years old, and from that time he had suffered from epilepsy, the attacks becoming more frequent as he advanced in age. He also stated to me that he had been treated by several specialists, but without benefit.

The analysis of the urine gave almost the same results as those obtained in case 1, with the exception that no trace whatever of albumen could be detected; bile, however, was found in considerable quantity.

The treatment resorted to in this case was 1-20 grain of pure phosphorus three times daily. The preparation used with this patient was an elixir of pure phosphorus, prepared by Dr. Chas. Mohr, of Mobile, Ala., and is one of the most elegant preparations I have ever seen. The young man, the time he came under my care, was also suffering from chronic malarial poisoning; so, in addition to the elixir of pure phosphorus, I gave him a pill, three times daily, as follows: Quiniæ sulph., ferri carbon (Vallet's mass) each gr. iss, Strychniæ sulph. gr. 1-20. At the end of three weeks these pills were discontinued, but the elixir of phosphorus was kept up for nearly three months longer. Since that time, more than three years ago, the patient has had no return of the epileptic attacks, his general health has much improved, and he has increased considerably in weight.

CASE NO. III.—Mr. G. aged about 35 years. Some 12 years previously he had a severe attack of cerebro-spinal mennigitis ; his recovery, so he informed me, had been slow and tedious. At the time I saw him, he was also suffering from an attack of malarial fever. He complained of constant dizziness, and there was a marked uncertainty of gait, the patient not having complete control over the co-ordination of movement. The analysis of the urine gave results similar to those obtained in case No. II. The treatment was the same as in No. II. The results in this case were highly gratifying to the patient, as well as myself. Once or twice afterwards he complained of slight symptoms, similar to those described above, but a resort to the phosphus remedy dispelled them.

It should be remembered that the various preparations of phosphorus are not here alluded to, the writer wishing only to refer to the value of phosphorus in its pure state.

Phosphorus enters largely into the constituents of the nervous tissues, the phosphorized fats forming fully 75 parts in 100 of their composition. it is, therefore, not unreasonable to assume, that, under its administration, the existing malnutrition of these tissues should be releived, and the nerve power increased by supplying the enormous nerve waste.

I am not prepared to state in what chemical condition pure phosphorus enters the circulation; this point has, as yet, not been fully determined. That it is readily absorbed, there seems to be little doubt. A part of it is taken up by the blood as uncombined phosphorus, owing to its solution by the alkalies of the intestinal fluids and fats, some portions, however, undergo various grades of oxidation ; some phosphide of hydrogen and phosphoric acid are also formed.

I do not claim pure phosphorus as a specific in locomotor ataxia or epilepsy, yet I think the drug should be given a fair trial. Negative results have so frequently been obtained, owing to the worthless character of the preparations used. Pills of so-called pure phosphorus are of no value, as they cannot be made or kept without undergoing oxidation. The only reliable mode of administration is in the shape of a solution.

There are certain conditions which should be carefully observed by the physician in the administration of pure phosphorus; he should use a great caution, and watch his patient closely. The drug should not be given just before, nor immediately after a meal, yet never on an entirely empty stomach. Upon the eructation of phosphoretted gases, the drug should be discontinued; also when it shows a tendency to produce diarrhœa; its administration must likewise be suspended when gastric disturbance occurs, as there is danger of producing a catarrhal state of the mucous membrane of the stomach.

Wegner (Virchow's Arch. June, 1872) maintain that the continued administration of pure phosphorus has a great tendency to increase the development of bone ; Kissel of St. Petersburg, however, finds this not to be the case. The views of these two observers are so positively opposed, that the statements of both should be taken with some degree of allowance. Nevertheless, it seems, from well authenticated cases, that there is great danger of inducing necrosis of the maxillary bones, if carous teeth exist; this point should, therefore, not be overlooked.

Two symptoms which should always warn the physician that he can proceed no further with the drug, is the appearance of albumen in the urine, and jaundice, however slight this last symptom may be. This latter condition is probably the result of resorption of the bile, causea by a catarrhal inflammation of the minute gall ducts, or by an inflammation of the mucous membrane of the common duct to such an extent as to offer an obstruction to the exit of the bile, or both.

When that peculiar condition of the skin known as phosphorodrosis, or phosphorescent perspiration follows the administration of pure phosphorus, the drug should be at once discontinued.

---

**Eminent Men of America.**—The Arlington Chemical Co. of Yonkers, N. Y., manufacturers of peptonoids and other digestives have favored the medical press with a neat little book under the above title, containing the biography and creditable portraits of Greeley, Beecher, Grant and other Americans who attained eminence in their several professions. Thanks for a copy; free on application.

